# Approach to the canine coxofemoral joint using an osteotomy of the deep gluteal muscle insertion for total hip replacements

**DOI:** 10.3389/fvets.2023.1224944

**Published:** 2023-08-31

**Authors:** Lauren Tardiani, Sarah Goldsmid, Otto Lanz

**Affiliations:** ^1^Animal Referral Hospital, Sydney, NSW, Australia; ^2^Department of Small Animal Clinical Sciences, Virginia-Maryland College of Veterinary Medicine, Blacksburg, VA, United States

**Keywords:** craniodorsal hip approach, osteotomy, deep gluteal, total hip replacement, coxofemoral, tenotomy

## Abstract

Canine total hip replacements (THR) are commonly performed using a craniolateral approach to the craniodorsal aspect of the hip which traditionally involves a partial deep gluteal tendon tenotomy (DGT). Performing an osteotomy of the insertion of the deep gluteal tendon has been utilized by some surgeons. Utilizing bone healing over tendon healing aims to improve post operative hip stability. To the authors’ knowledge, this is the first published description of the novel deep gluteal osteotomy (DGO) approach. It differs from a greater trochanteric osteotomy as the latter involves an osteotomy of both the deep and middle gluteal insertions. DGT and the novel DGO approach were performed in ten medium to large breed cadavers on contralateral limbs. The surface area of acetabular exposure was measured in contralateral limbs following the approaches and the iatrogenic damage to the deep gluteal, middle gluteal and vastus lateralis muscles following femoral reaming was visually graded (none, mild, moderate or severe) based on muscle proportion damaged. There was no statistically significant difference (*p* = 0.8223) between the surface area of acetabular cartilage exposed by each approach with the mean surface area for the DGO approach being 2.99 cm^2^, whilst the mean surface area for the DGT was 2.97 cm^2^. In 80% of cadavers, the DGO approach achieved lower muscle damage following retraction and femoral reaming compared to the DGT approach for the middle gluteal and deep gluteal muscles (*p* = 0.0073). In all cadavers, overall muscle damage was lower for the DGO approach compared to the DGT approach (*p* = <0.001). There was no difference in vastus lateralis damage between procedures. The DGO approach may be a reasonable alternative to the DGT for approaching the hip joint and femur for Zurich THR as it provides similar exposure to the acetabulum with less muscle damage. It relies on more reliable osseous healing compared to tendon healing. Reduced muscle damage may be important for postoperative hip stability following THR. Further studies are required to biomechanically assess the strength of DGO repair compared to DGT repair as well as a case series documenting clinical outcomes.

## 1. Introduction

The canine coxofemoral joint is surgically approached for a variety of procedures including total hip replacement (THR), open hip reduction and stabilisation following acute/traumatic coxofemoral luxation, open reduction and internal fixation of femoral head and neck fractures, and femoral head and neck excisional arthroplasty (1). The craniolateral approach to the craniodorsal aspect of the hip, including a partial deep gluteal tendon tenotomy, is traditionally used (1). When additional exposure is required, an osteotomy of the greater trochanter exposes the caudodorsal hip by elevating the insertions of the middle and deep gluteal muscles (1, 2).

In human THRs, the extent of soft tissue dissection and the importance of soft tissue tension in maintaining hip stability have long been recognized (3–5). Further, a relationship has been identified between the surgical approach and post operative hip stability as documented by post operative luxation rates following THR in humans (3–7). Whilst these factors have received minimal attention in the veterinary literature, Hayes et al. (8) found that pre-existing subluxation/soft tissue laxity is a significant risk factor for post operative luxation following canine THR.

Some veterinary surgeons have raised concerns regarding the potential impact on hip rotational stability following the standard approach, despite repair of deep gluteal partial tenotomy. The described closure of the standard craniolateral hip approach includes repair of the deep gluteal tenotomy by placement of one or two mattress sutures or a pulley suture (1). A running cruciate suture pattern is also a common repair technique for both Kyon Zurich Cementless THR and Biomedtrix THR [(9), 2023 personal communication with Kowaleski, Michael regarding closure following Biomedtrix THR, unreferenced]. A repaired tendon has reduced tensile strength compared to an intact tendon and Markel et al. (10) found tenotomy repair of the middle gluteal in a canine model, showed significantly reduced strength compared to non-tenotomized controls. Despite widespread use of the standard hip approach by veterinary surgeons, there is minimal literature regarding the strength of the repair and closure, and none, to the authors’ knowledge, regarding effect on patient outcomes. No evidence-based recommendations regarding location and size of the tenotomy were found by the authors of this study.

Adequate exposure minimises excessive force when retracting muscles and hence direct injuries on the muscles (1). It follows that muscle damage may reflect ease of accessibility of the approach. In the authors’ experience, an osteotomy of the insertion of the deep gluteal muscle, rather than deep gluteal tendon partial tenotomy, allows improved access to the hip joint for femoral and acetabular reaming during Kyon THR, whilst concurrently enabling less traumatic retraction of the middle gluteal, deep gluteal and vastus lateralis muscles. Others have noted muscle damage associated with total hip replacements (11). This may also impact hip stability during the post-operative period. Acknowledgement of this damage and its potential consequences has recently resulted in some surgeons choosing to perform a complete or almost complete deep gluteal tenotomy rather than the traditional partial tenotomy when performing THRs thus allowing better exposure and less damage to the deep gluteal muscle during reaming (12).

Approaching the canine coxofemoral joint using an osteotomy of the insertion of the deep gluteal muscle, rather than deep gluteal tendon partial tenotomy, ensures the deep gluteal tendon strength is maintained to aid in postoperative hip stability. The authors also propose that this approach minimizes muscle damage related to retraction for THR reaming likely due to improved access.

Additionally, the osteotomy closure relies on bone healing rather than tendon healing, which has been well documented to be more reliable (13). Furthermore, bone to bone fixation is typically stronger than tendon repair and Markel’s et al. (10) study found bone to bone fixation following greater trochanter osteotomy was stronger than all tendon repair techniques and the only repair comparable in strength to the intact middle gluteal tendon.

To the authors’ knowledge, there are no published descriptions of the osteotomy of the deep gluteal insertion approach. The objectives of this study are to describe the osteotomy technique and to (1) quantitatively compare the acetabular exposure following DGO (deep gluteal insertion osteotomy) with that of a DGT (deep gluteal tendon partial tenotomy), and (2) to compare iatrogenic muscular damage to deep gluteal muscle, middle gluteal muscle and vastus lateralis muscles following femoral reaming for the Zurich Cementless THR in cadavers. We hypothesised that DGO would provide at least equivalent exposure of the acetabulum and reduced iatrogenic muscular damage related to retraction and femoral reaming.

## 2. Materials and methods

### 2.1. Specimen collection

Client-owned dogs were euthanized for reasons unrelated to the study and owners consented to donation and research use. The thawed skeletally mature canine cadaveric whole specimens (*n* = 12) with no known history of previous pelvic limb pathology or surgery, were used in accordance with the Animal Ethics Committee’s policy on use of cadavers. Cadavers were medium to large breed dogs weighing between 19.1 and 47.4 kg.

### 2.2. Initial screening and specimen preparation

Sex, body weight and BCS (1–9) were recorded for each cadaver, though age and reproductive status were not as these were not available. Cadavers were assessed for symmetry of hindlimbs by palpation for orthopaedic abnormalities, radiographic assessment, and thigh circumference measurements. Thigh circumference was measured for each cadaver according to previously published recommendations (14). Cadavers were excluded if initial screening revealed asymmetry in muscle mass or asymmetric orthopaedic disease. Presence of osteoarthritis did not result in exclusion provided disease was not asymmetric. The left hip was approached first and cadavers were randomly allocated by blinded selection to either a left sided DGO or DGT approach (50% of the choices were DGO and 50% were DGT). The alternative approach was performed on the contralateral hip. DGO and DGT were performed by an experienced small animal surgery specialist with experience performing both techniques.

### 2.3. Approach description

For all specimens, the approach from skin incision to exposure of the deep and middle gluteal muscles was performed as described previously (1). To maintain uniformity, the skin incision was first measured and drawn with a marker. It was centred over the proximal tip of the greater trochanter and extended equidistant 30% of the thigh length proximally and distally. The superficial leaf of the fascia lata was incised along the cranial border of the biceps femoris muscle then retraction of this muscle exposed deeper tissues. An intermuscular incision was made along the cranial border of the superficial gluteal muscle and continued distally through the deep leaf of the fascia lata. Cranial retraction of fascia lata and tensor fascia lata muscle and caudal retraction of the biceps muscle exposed the middle gluteal and vastus lateralis muscles.

#### 2.3.1. Partial deep gluteal tenotomy approach

This was performed as described previously (1). Dorsal retraction of the middle gluteal muscle exposed the deep gluteal muscle and tendon. The tenotomy was made between 5–10 mm from its insertion and across approximately 50% of the deep gluteal tendon diameter at this level as judged visibly and palpably by the surgeon. Capsulotomy was performed in a T-shape by incising along its attachment to the femoral neck, then extending dorsally.

Capsulotomy incision was extended distally along the origin of the vastus lateralis muscle along the neck and lesser trochanter and this origin was elevated distally.

The round ligament was transected using a Hatt spoon, the femoral head luxated and a femoral head ostectomy was performed as described for the Zurich Cementless total hip replacement (15). This ostectomy involves removing the ridge of bone between the greater trochanter and the femoral head to a level just distal to the femoral head, whilst preserving the calcar. An oscillating saw is then utilized to perform the femoral head ostectomy.

#### 2.3.2. Deep gluteal insertion osteotomy approach

**Figure 1:**
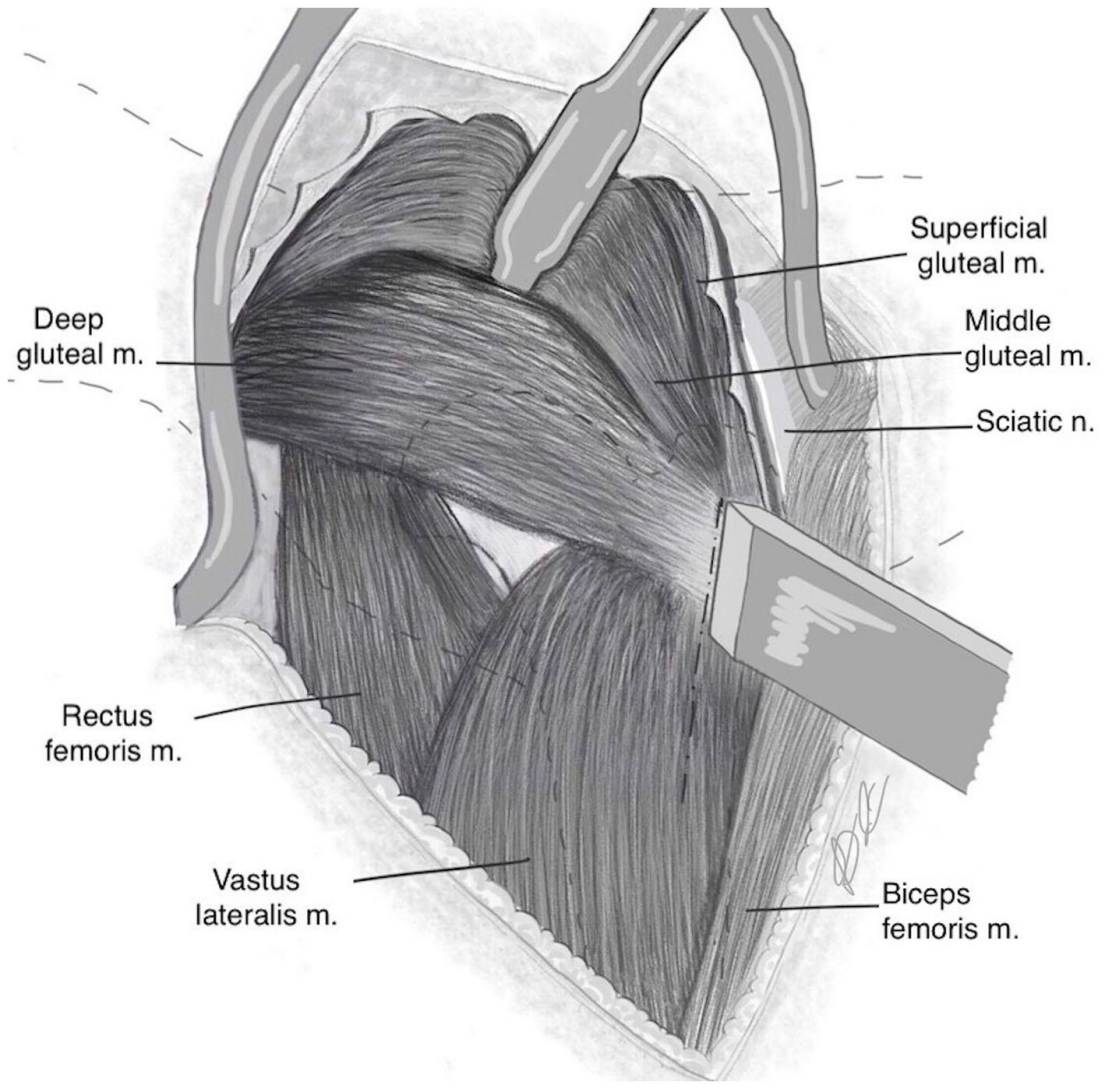
Following dorsal retraction of the middle gluteal muscle, a Hohman retractor is placed dorsal to the deep gluteal muscle to help define the dorsal extent of the muscle. The osteotomy of the cranial third of the width of the greater trochanter is performed to include the deep gluteal tendon insertion and a small portion of vastus lateralis origin. The dashed line shows osteotomy and extension into vastus lateralis muscle along the muscle fibres, preserving the insertion of the middle gluteal muscle.

Dorsal retraction of the middle gluteal muscle exposed the deep gluteal muscle and its insertion onto the greater trochanter (Figure 1). A Hohmann retractor was placed dorsal to the deep gluteal tendon to help define dorsal extent of muscle. The deep gluteal insertion lies adjacent and distomedial to that of the middle gluteal insertion.

**Figure 2:**
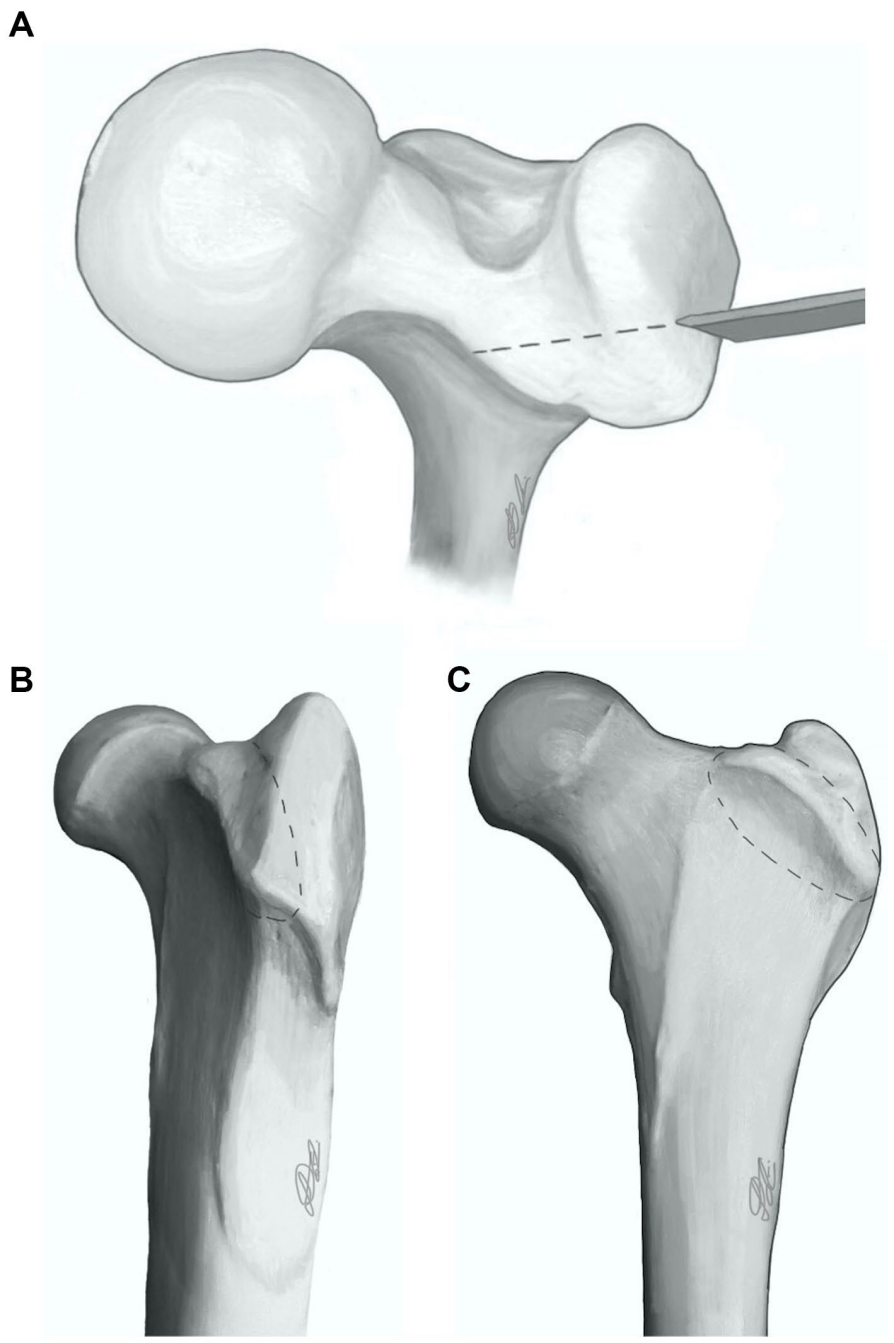
Proximal femur showing deep gluteal osteotomy (dashed line) configuration from three perspectives: dorsal **(A)**, craniolateral **(B)**, and cranial **(C)**.

With the hip internally rotated, the width of the greater trochanter was identified. In order to delineate the osteotomy line, the cranial third of the greater trochanter was approximated (Figures 2A,B). This captured the deep gluteal insertion, whilst excluding the middle gluteal insertion. The line of the osteotomy was first marked with a scalpel from its proximal extent distally through the vastus lateralis muscle along its fibres.

The osteotomy was performed with an osteotome and mallet in a caudolateral to craniomedial direction (Figures 2A–C) and included the entire deep gluteal muscle insertion and a small portion of the vastus lateralis muscle. An oscillating saw may also be used.

**Figure 3:**
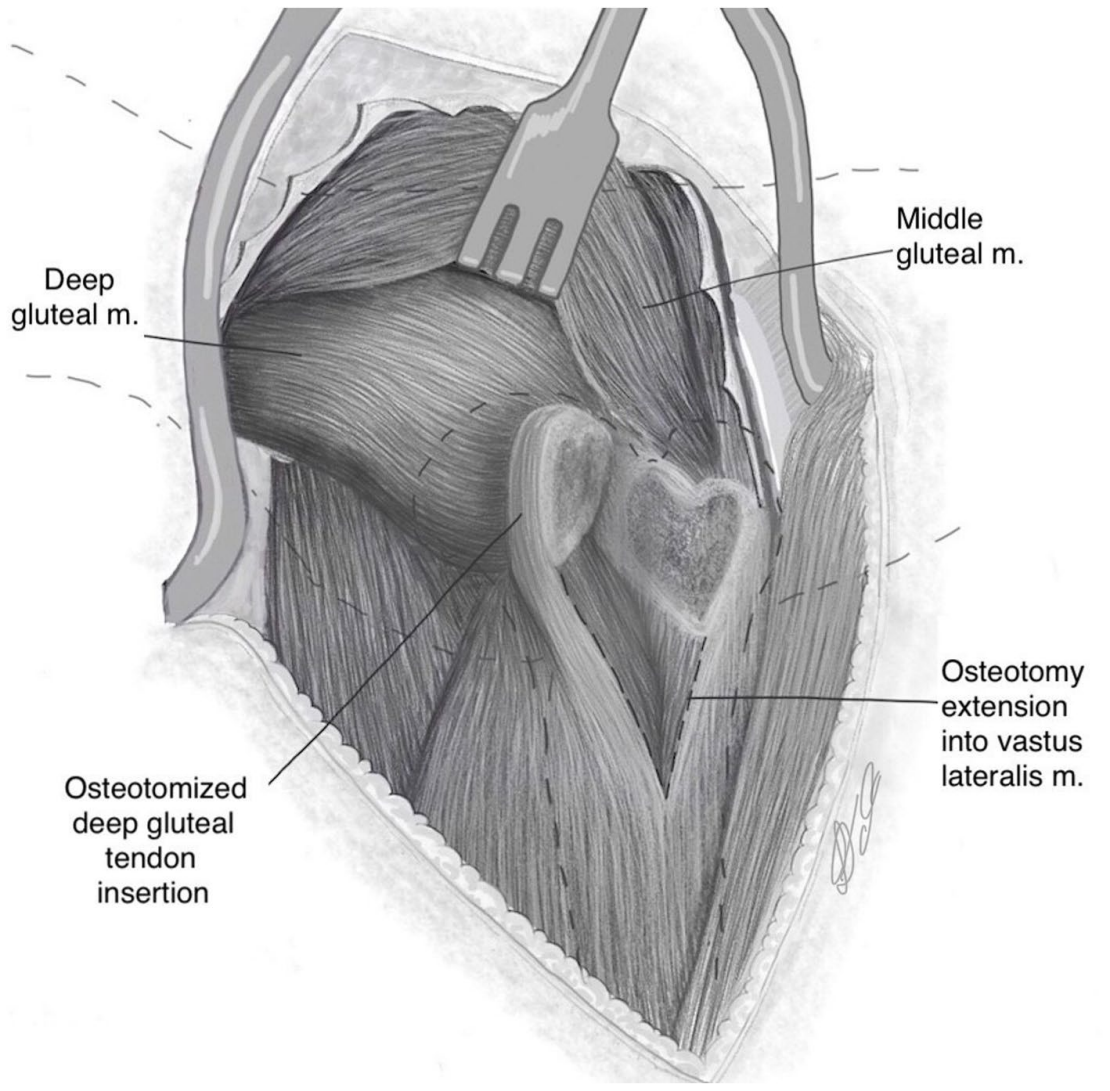
Following osteotomy and extension into vastus lateralis muscle, the osteotomised bone is reflected cranially revealing a “heart-shaped” osteotomy surface.

The deep gluteal and part of the vastus lateralis muscle with their trochanteric attachments were retracted cranially (Figure 3). In doing so, soft tissue attachments were freed dorsally. Note that elevation of the origin of the vastus lateralis muscle is not necessary.

**Figure 4:**
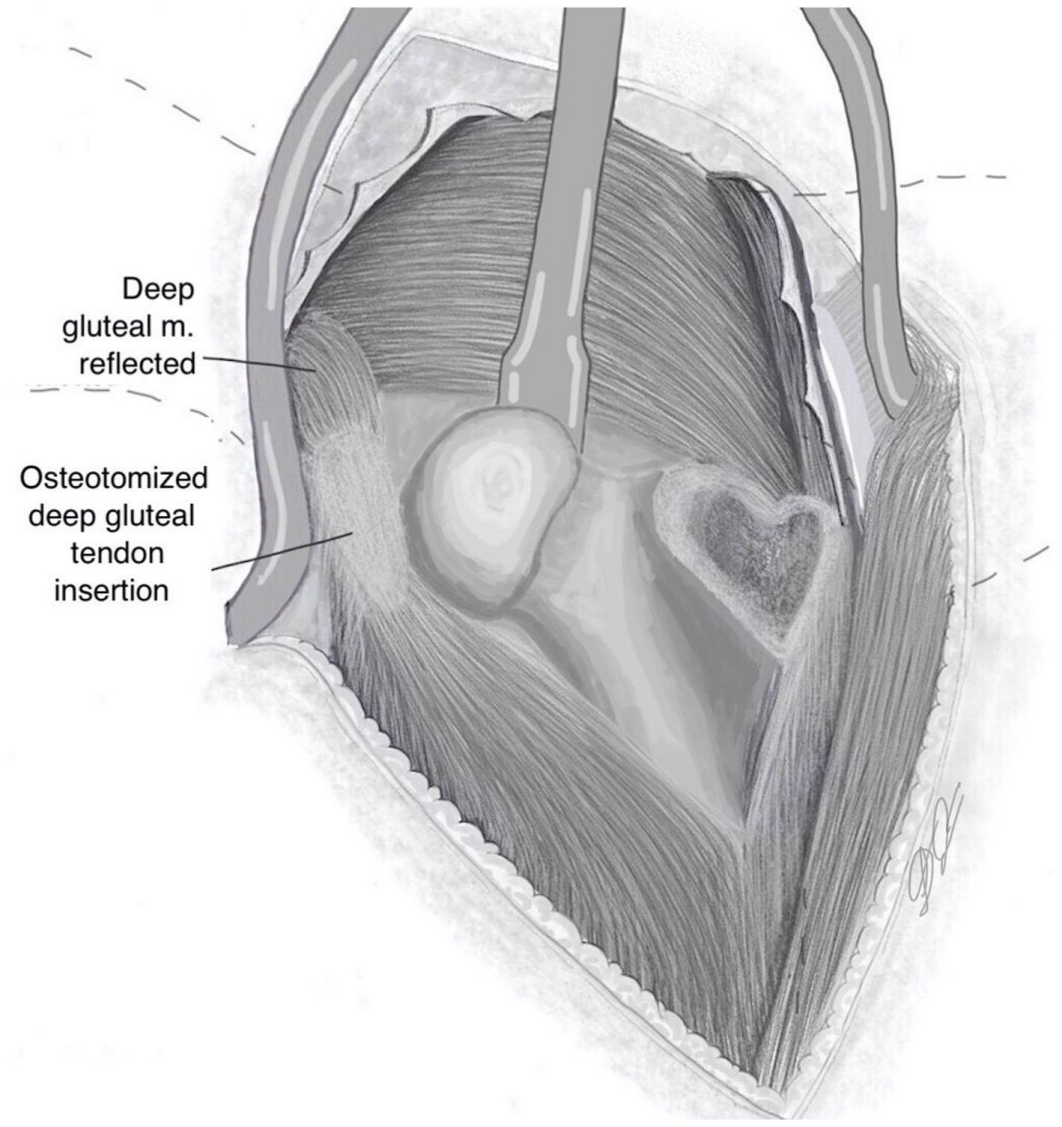
Exposure of proximal femur following cranial retraction of osteotomised bone with its muscular attachments, capsulotomy, femoral head disarticulation and external rotation of femur.

The limb was externally rotated and capsulotomy was then performed by incising along its attachment to the femoral neck, then extended dorsally, creating a T-shape. The round ligament was transected, the femoral head luxated (Figure 4) and a femoral head ostectomy was performed as previously described for the Zurich Cementless total hip replacement (15).

**Figure 5:**
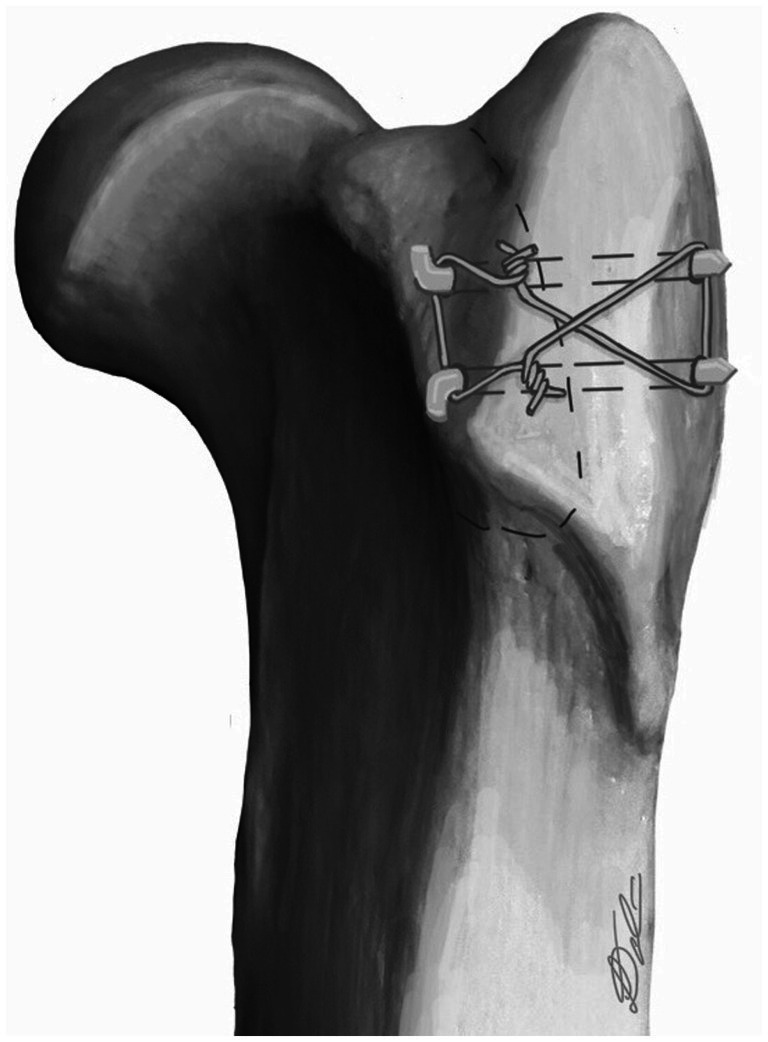
Proximal femur showing DGO repair technique with 2 pins and a tension band.

**Figure 6:**
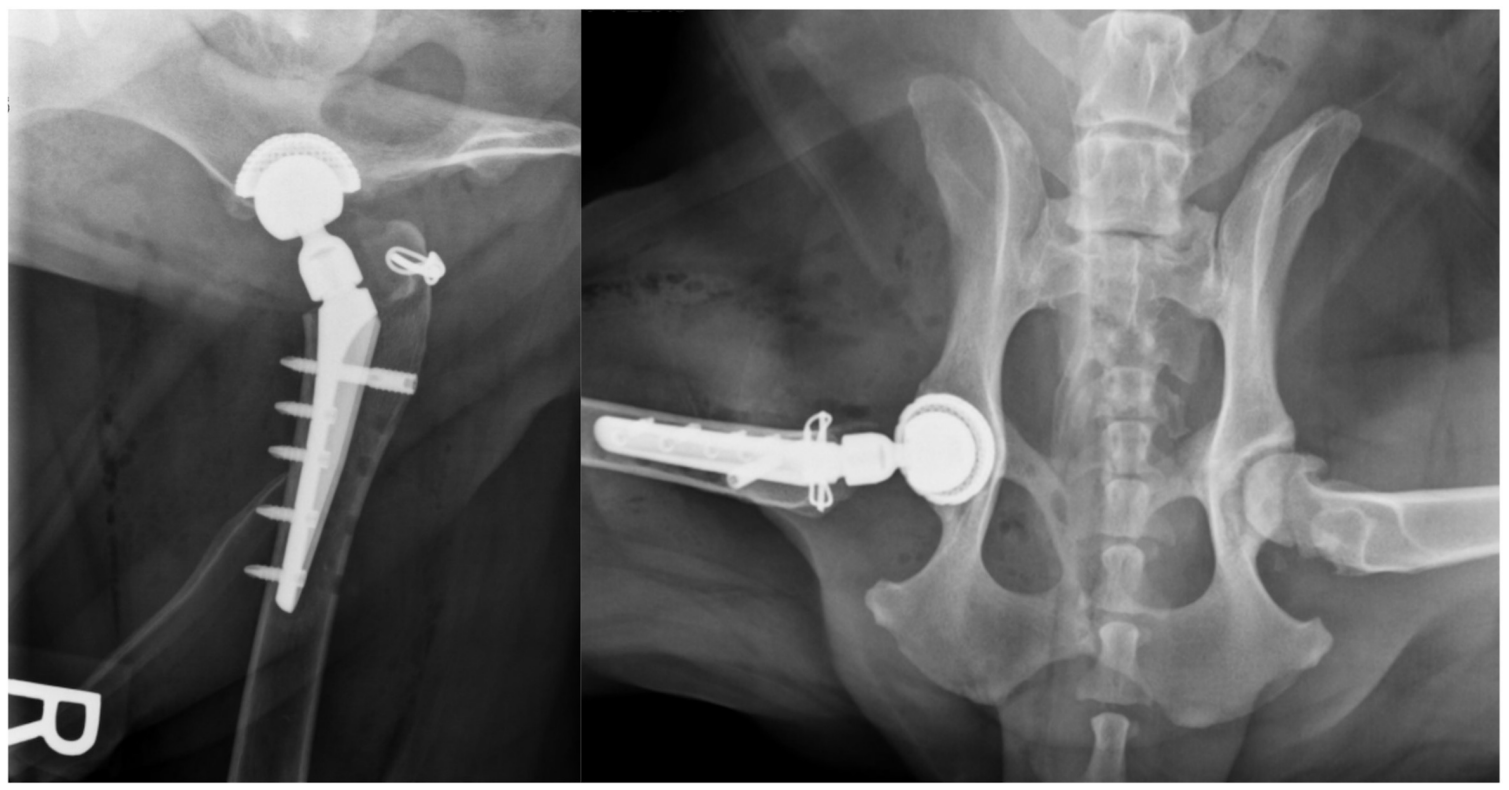
Post operative radiographs showing author’s post DGO repair when the approach was used for a total hip replacement.

Repair of the osteotomy and tenotomy were not performed as part of this study. Several repair methods for the DGO have been used by the authors. The favoured repair method, based on clinical experience, is to place two threaded pins and one tension band wire across the osteotomy following reduction and compression with point-to-point forceps (Figures 5, 6).

### 2.4. Procedures—femoral reaming

Following DGO or DGT and femoral head ostectomy, the femur was externally rotated with stifle pointing laterally. Two self-retaining Gelpi retractors were placed across the soft tissues and a Hohmann retractor was positioned beneath the proximal femur to access the femoral canal for femoral reaming. Femoral reaming was performed for the appropriately sized Zurich Cementless THR femoral stem determined from the radiographs.

### 2.5. Measures of outcome

#### 2.5.1. Acetabular exposure

**Figure 7:**
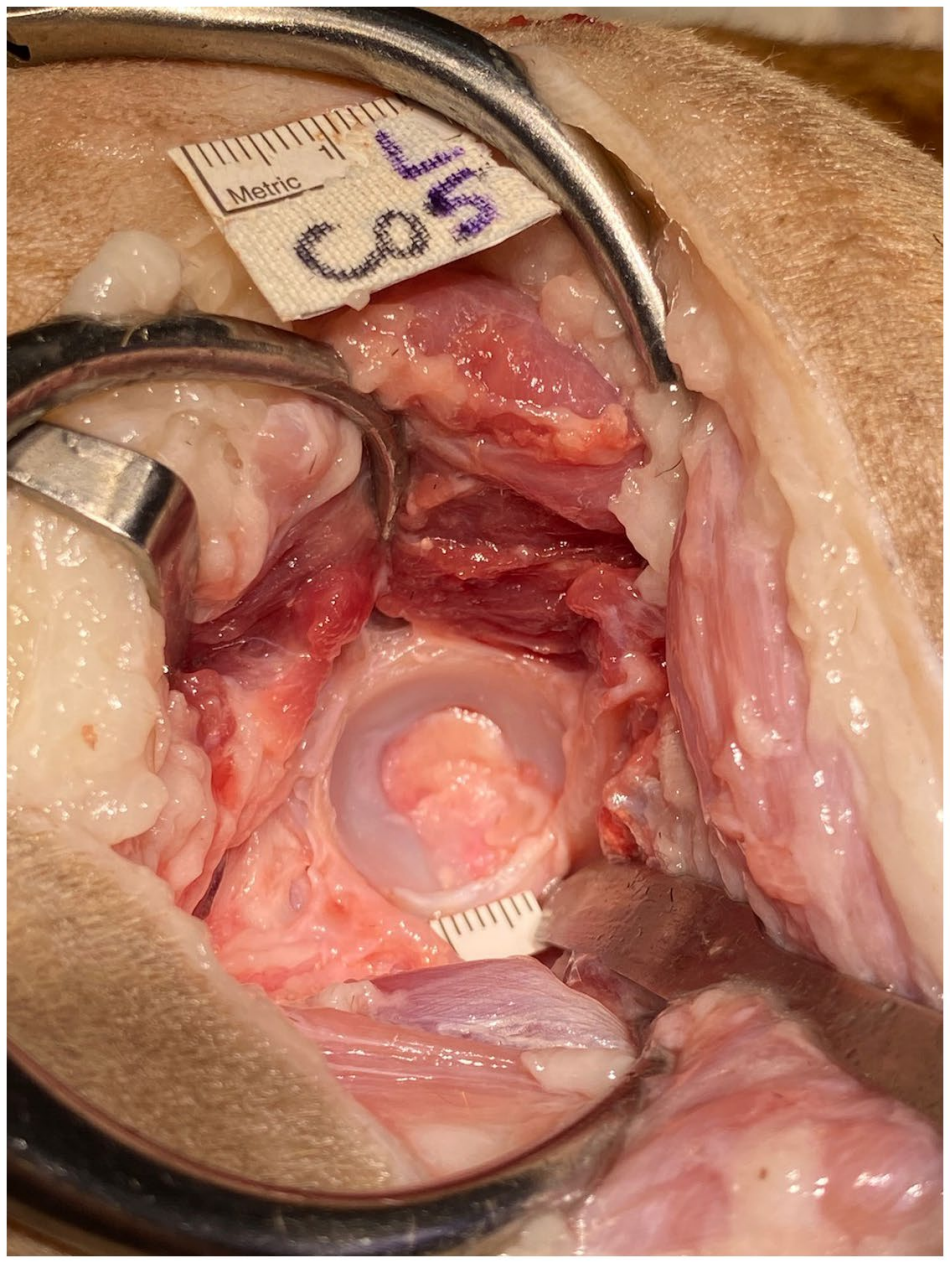
Exposed left acetabulum of cadaver 5. Retractors were positioned for optimal exposure and a 5 mm ruler was placed in a repeatable position, at the level of the acetabulum at the ventral aspect.

Retractors were repositioned to achieve optimal exposure and a 5 mm ruler was placed in a repeatable position, at the level of the acetabulum at the ventral aspect (Figure 7). Multiple digital photographs (iPhone 11 Pro, Apple, CA) were taken from the surgeon’s perspective. The three best photographic views of the acetabulum were selected. These calibrated images were then imported into a computer program, ImageJ (National Institutes of Health, Bethesda, MD, United States), and the exposed acetabular surface area was calculated. The exposed area of the acetabulum was outlined manually, and a known distance (5 mm ruler) and number of pixels was converted to surface area. Each of the selected images were measured three times and the mean of these was recorded as the surface area of acetabular exposure in cm^2^ for that hip.

#### 2.5.2. Iatrogenic muscle damage following retraction and femoral reaming

**Figure 8:**
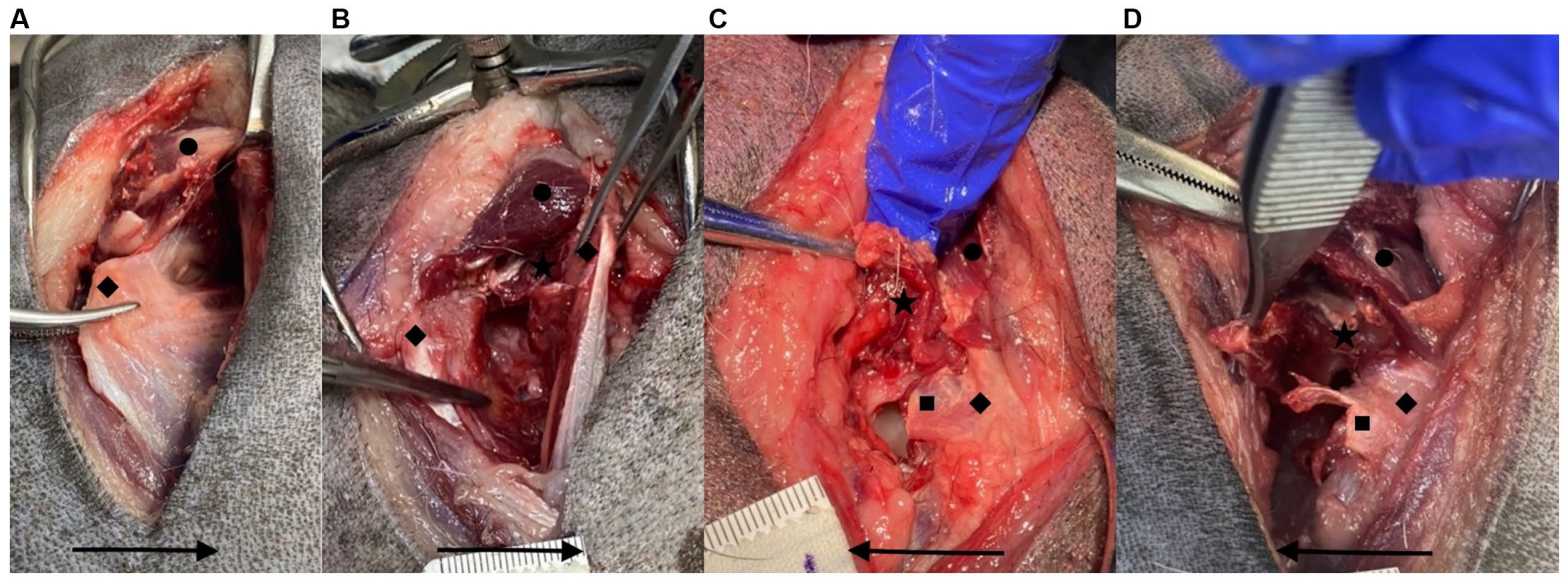
Examples of no **(A)**, mild **(B)**, moderate **(C)**, and severe **(D)** iatrogenic muscle damage for the deep gluteal muscle. ★ = iatrogenic deep gluteal muscle damage, ◆ = greater trochanter, ● = middle gluteal muscle, ■ = deep gluteal tenotomy. Arrow points in a cranial direction.

Iatrogenic muscular damage was grossly visually assessed for the deep gluteal, middle gluteal and vastus lateralis. Each muscle was assessed independently and either no, mild, moderate or severe damage recorded for each (Figure 8). Visible muscle tear/defect that appeared to affect less than 25% of muscle diameter was considered “mild”; greater than 25% but less than 50% of the muscle diameter was “moderate”; whilst greater than 50% was considered “severe.” When visible disruption to integrity of the muscle was not identified, this was considered “no damage.” Overall, left or right side was assessed as having sustained greater muscular damage than the contralateral side. Damage directly associated with the tenotomy was excluded from this assessment as was the longitudinal incision in the vastus lateralis associated with DGO.

### 2.6. Statical analysis

Statistical analysis compared DGO to DGT for the following factors: (1) level of acetabular exposure measured as surface area cm^2^; and (2) degree of muscle damage for middle gluteal, deep gluteal, vastus lateralis muscles, as well as total damage was assessed. As the level of exposure was a continuous variable, a paired *t*-test was used to compare the surface area achieved with each approach. Due to minimal variability in the level of muscular damage achieved by the DGO approach the gluteal damage was analyzed by analyzing the proportion of times the DGO approach achieved lower damage compared to the DGT approach against the proportion of times DGO did not achieve lower damage. All statistical analyses were performed using Stata Statistical Software: Release 17 (StataCorp LLC., College Station, TX).

## 3. Results

### 3.1. Specimens

Twelve specimens were acquired and underwent initial screening with two cadavers excluded for asymmetry (severe osteoarthritis in one hip only in one cadaver, and asymmetric muscle mass of unknown origin in another). Ten medium to large breed canine cadavers (4 females, 6 males; weight range = 19.1 to 47.4 kg; mean body weight = 32.8 kg) were included in the study.

### 3.2. Exposure

There was no statistically significant difference between the level of exposure to the acetabulum achieved by the DGO and DGT approaches (*p* = 0.8223). The mean surface area for the DGO approach was 2.99 cm^2^, whilst the mean surface area for the DGT was 2.97 cm^2^.

### 3.3. Iatrogenic muscle damage following retraction and femoral reaming

In 8/10 cadavers the DGO approach achieved less damage compared to the DGT approach for the middle gluteal muscle which was a statistically significant difference (*p* = 0.0073). Equally, in 8/10 cadavers the DGO approach achieved less damage compared to the DGT approach for the deep gluteal muscle (*p* = 0.0073). There was less damage to the vastus lateralis muscles compared to other muscles, with 60% of all hips sustaining no damage to this muscle. Vastus lateralis damage was greater in both DGO and DGT an equal number of times and so not statistically significant (*p* = 1.0). For all the cadavers (10/10) the overall muscle damage was lower for the DGO approach compared to the DGT approach which was statistically significant (*p* = <0.001).

## 4. Discussion

This paper describes a novel surgical approach to the canine coxofemoral joint. The cadaver study component supported our hypothesis that DGO allowed comparable exposure to the DGT approach. Less iatrogenic muscle damage was observed to the deep and middle gluteal muscles, due to femoral reaming and retraction, although not the vastus lateralis muscle.

The DGO was first performed to avoid complications related to healing of the deep gluteal tendon, and hence, minimise post operative instability. The authors routinely perform this approach for THRs as they feel it gives improved access for femoral and acetabular reaming with improved postoperative stability.

Exposure of the hip is difficult to quantify due to its depth and three-dimensional structure as well as the importance of surrounding soft tissue retraction in the accessibility. Measurement of the acetabular cartilage surface area was used as a measure of exposure and was not found to be statistically different between the DGO and DGT approaches. An additional outcome measure was used to assess the accessibility for femoral reaming. Iatrogenic muscular damage following retraction for femoral reaming was found to be lower for all cadavers when the DGO approach was used. When deep gluteal and middle gluteal muscles were assessed individually, the DGO approach resulted in statistically significant reduced muscle damage in 8/10. There was no difference between approaches when the vastus lateralis muscle was assessed. It is important to note that the assessment was only of muscle damage secondary to retraction for femoral reaming, and did not assess damage to muscles due to the actual approach. The vastus lateralis origin is elevated in the DGT approach, and there is a longitudinal incision in the muscle associated with the DGO approach.

Previous human studies compared the exposure of different surgical approaches by measuring exposed osseous surface area via similar methodology to this study (16–18). Calibrated digital photographs were taken from the surgeon’s perspective and analysed in ImageJ software by comparing a known length to number of pixels. Measurement of acetabular area helped to establish that the DGO provides the surgeon with at least equivalent exposure of the acetabulum as DGT. Given the concave nature of the acetabular surface it may have been a limitation to measure this with a 2D image.

When performing a THR, it is not only the osseous exposure that is important but also the ability to access these bone structures with instruments. Given the depth of the hip within the musculature, it follows that improved ability to retract the soft tissues would be expected to correlate with better access for performing femoral or acetabular reaming. Optimal exposure and access are beneficial for reducing the forces required to retract musculature. Anecdotally, it is recognized by surgeons that at the end of a THR where DGT is used, due to the significant manipulation required to access relevant anatomy, the musculature (particularly the deep and middle gluteal muscles) becomes notably damaged. This observation was supported by our study findings in that less muscular damage occurred with DGO, which may correlate with improved access for femoral reaming.

The reduction in muscle damage with DGO is not only important as an indication of ease of access, but the consequences of muscle damage are likely to be important for post operative hip stability. The deep and middle gluteal muscles function to extend the hip with some abduction of the femur, and on weight bearing they medially rotate the hip whilst preventing lateral rotation (19). It follows that functional preservation of these structures during the surgical approach is likely to contribute to hip stability postoperatively (3–8).

Furthermore, the degree of muscular damage assessed did not consider the damage from the partial deep gluteal tenotomy itself, nor the elevation of vastus lateralis origin. A repaired tenotomy is significantly weaker than its intact equivalent and failure tends to occur due to suture pull out (10, 20). Numerous suture patterns are proposed for closure of a tendon, however the described DGT closure includes one or two mattress sutures, a pulley suture or a continuous cruciate pattern. Markel et al. (10) used a canine cadaver model of an isolated middle gluteal muscle to compare tenotomy closure, tendon to bone closure, osteotomy repair, and an intact control. The bone-to-bone repair was the only repair that did not have less tensile strength and stiffness than the control. Across the four tendon repairs there was no significant difference in tensile strength or stiffness. The tendon to tendon repair methods achieved a stiffness that was 32% that of the control and an ultimate load that was 39% of the control. It should be noted that this study compared complete tenotomies rather than partial tenotomies, and specifically addressed the middle gluteal muscle.

Implant failure is a potential complication of DGO which could theoretically result in external rotation of the coxofemoral joint and predispose to luxation. The recommended repair technique of the DGO is based on the authors’ experience. Biomechanical assessment as well as clinical outcomes are beyond the scope of this paper. In the authors’ personal experience, in the small number of cases with DGO failure, external rotation of the limb has been noted, though surgical revision has not been necessary. In the authors’ experience the DGO failure has not occurred with the pins and tension band repair technique described. An intact middle gluteal insertion may contribute to stability compared to a complete greater trochanter osteotomy and further studies are necessary to investigate this.

We acknowledge there are a number of limitations of this study. There was potential for bias in assessment of the degree of muscular damage with each technique, however it would have been difficult to blind an observer as the osteotomy is in the field of view. There is a need for biomechanical studies to compare the strength of DGO to DGT and greater trochanter osteotomy repairs. This study specifically assessed the approach and its application for Kyon THR implants and did not assess relative benefits for different types of THR implant systems. In dogs, it is not known if a failed deep gluteal tendon repair influences hip stability and clinical outcomes postoperatively. *In vivo* muscle tissue may behave differently to cadaver preparations and a prospective clinical study is warranted. The population size is small in this study, however it is comparable to those used in human studies assessing exposure of various approaches. The variation in cadaver sizes may also be considered a limitation and the study does not directly assess ease of acetabular or femoral reaming for each approach.

The DGO technique has been shown to result in equal acetabular cartilage exposure as the standard approach to the canine coxofemoral joint for Kyon THR using a DGT. The DGO approach appears to improve accessibility to the proximal femur for reaming during Kyon THRs resulting in less intraoperative muscle damage. Further studies are required to biomechanically assess the DGO repair compared to DGT. A case series showing the clinical outcomes of coxofemoral joint procedures utilizing the DGO compared with the DGT is indicated.

## Data availability statement

The raw data supporting the conclusions of this article will be made available by the authors, without undue reservation.

## Ethics statement

Ethical approval was not required for the studies involving animals in accordance with the local legislation and institutional requirements because the animals were euthanized for reasons unrelated to the study. Written informed consent was obtained from the owners for the participation of their animals in this study.

## Author contributions

LT and SG contributed to conception and design of the study and performed the cadaver study. LT analysed the images in ImageJ, wrote the first draft of the manuscript, and illustrated Figures 1-5. All authors contributed to the article and approved the submitted version.

## Conflict of interest

The authors declare that the research was conducted in the absence of any commercial or financial relationships that could be construed as a potential conflict of interest.

## Publisher’s note

All claims expressed in this article are solely those of the authors and do not necessarily represent those of their affiliated organizations, or those of the publisher, the editors and the reviewers. Any product that may be evaluated in this article, or claim that may be made by its manufacturer, is not guaranteed or endorsed by the publisher.

## Abbreviations

THR: Canine total hip replacements
DGT: Partial deep gluteal tendon tenotomy
DGO: Deep gluteal osteotomy

## References

[1] Johnson KA. Piermattei’s atlas of surgical approaches to the bones and joints of the dog and cat. 5 Pennsylvania, PA: Saunders (2013), 340–345 p.

[2] Silveira CJ Saunders WB. Greater trochanter osteotomy as a component of cementless total hip replacement: five cases in four dogs. Vet Surg. (2021) 51:303–10. doi: 10.1111/vsu.13742, PMID: 34724235

[3] Petis S Howard JL Lanting BL Vasarhelyi EM. Surgical approach in primary total hip arthroplasty: anatomy, technique and clinical outcomes. Can J Surg. (2015) 58:128–39. doi: 10.1503/cjs.007214, PMID: 25799249 PMC4373995

[4] Dargel J Oppermann J Brüggemann GP Eysel P. Dislocation following total hip replacement. Dtsch Arztebl Int. (2014) 111:51–2. doi: 10.3238/arztebl.2014.0884 PMC4298240 25597367

[5] Woo RYG Morrey BF. Dislocations after total hip arthroplasty. J Bone Jt Surg. (1982) 64:1295–306. doi: 10.2106/00004623-198264090-00004 7142237

[6] Sundaram K Siddiqi A Kamath AF Higuera-Rueda CA. Trochanteric osteotomy in revision total hip arthroplasty. EFORT Open Rev. (2020) 5:477–85. doi: 10.1302/2058-5241.5.190063, PMID: 32953133 PMC7484712

[7] Masonis JL Bourne RB. Surgical approach, abductor function, and total hip arthroplasty dislocation. Clin Orthop Relat Res. (2002) 405:46–53. doi: 10.1097/00003086-200212000-00006 12461355

[8] Hayes GM Ramirez J Langley Hobbs SJ. Does the degree of preoperative subluxation or soft tissue tension affect the incidence of postoperative luxation in dogs after total hip replacement? Vet Surg. (2011) 40:6–13. doi: 10.1111/j.1532-950X.2010.00754.x 21070265

[9] Vezzoni A. Zurich cementless total hip replacement surgical technique Vol 1.3. Kyon Veterinary Surgical Products (2014).

[10] Markel MD Rock MG Bergenthal DS Young DR Vanderby R Chao EYS. A mechanical comparison of gluteus medius attachment methods in a canine model. J Bone Jt Surg. (1993) 11:457–61. doi: 10.1002/jor.1100110320 8326454

[11] Wylie KB DeYoung DJ Drost WT DeYoung BA. The effect of surgical approach on femoral stem position in canine cemented total hip replacement. Vet Surg. (1997) 26:62–6. doi: 10.1111/j.1532-950X.1997.tb01464.x, PMID: 9123815

[12] Kowaleski M. Personal communications. Medford, MA: (2023).

[13] Cottrell JA Turner JC Arinzeh TL O’Connor JP. The biology of bone and ligament healing. Foot Ankle Clin. (2016) 21:739–61. doi: 10.1016/j.fcl.2016.07.017 27871408

[14] McCarthy DA Millis DL Levine D Weigel JP. Variables affecting thigh girth measurement and observer reliability in dogs. Front Vet Sci. (2018) 5:1–8. doi: 10.3389/fvets.2018.00203, PMID: 30214905 PMC6125300

[15] Hummel D. Zurich cementless total hip replacement. Vet Clin North Am Small Anim Pract. (2017) 47:917–34. doi: 10.1016/j.cvsm.2017.02.004 28442161

[16] Lichstein PM Kleimeyer JP Githens M Vorhies JS Gardner MJ Bellino M. Does the Watson–Jones or modified Smith–Petersen approach provide superior exposure for femoral neck fracture fixation? Clin Orthop Relat Res. (2018) 476:1468–76. doi: 10.1097/01.blo.0000533627.07650.bb 29698292 PMC6437565

[17] Jost B Benninger E Erhardt JB Külling FA Zdravkovic V Spross C. The extended medial elbow approach-a cadaveric study. J Shoulder Elb Surg. (2015) 24:1074–80. doi: 10.1016/j.jse.2015.03.013, PMID: 25940381

[18] Singh K Weitlich JD Zitsch BP Schweser KM Cook JL Crist BD. Which surgical approach provides maximum visualization and access for open reduction and internal fixation (ORIF) of femoral neck fractures? Injury. (2022) 53:1131–6. doi: 10.1016/j.injury.2021.11.023, PMID: 34809924

[19] Evans H de Lahunta A. Miller’s anatomy of the dog. 4 St. Louis, Missouri: Elsevier (2013) 384–385

[20] Giuseffi SA Wongtriratanachai P Omae H Cil A Zobitz ME An KN. Biomechanical comparison of lesser tuberosity osteotomy versus subscapularis tenotomy in total shoulder arthroplasty. J Shoulder Elb Surg. (2012) 21:1087–95. doi: 10.1016/j.jse.2011.07.008, PMID: 21982350

